# The impact of child poverty on brain development: does money matter?

**DOI:** 10.1590/1980-5764-DN-2022-0105

**Published:** 2023-08-07

**Authors:** Diogo Macedo Feijó, Jackson Frederico Pires, Regiane Maria Ribeiro Gomes, Ettore José Filippi Carlo, Tayenne Nélly de Lucena Viana, Jacqueline Rodrigues Magalhães, Amanda Cristine Trevisan Santos, Laís Damasceno Rodrigues, Leandro Freitas Oliveira, Júlio César Claudino dos Santos

**Affiliations:** 1 Universidade Estadual da Paraíba, Paraíba PB, Brazil.; 2 Universidade Nove de Julho, Bauru SP, Brazil.; 3 Universidade Estácio de Sá, São Paulo SP, Brazil.; 4 Faculdades Metropolitanas Unidas, São Paulo SP, Brazil.; 5 Universidade de Fortaleza, Fortaleza CE, Brazil.; 6 Faculdade Alpha, Recife PE, Brazil.; 7 Instituto Israelita Albert Einstein, São Paulo SP, Brazil.; 8 Universidade Católica de Brasília, Brasília DF, Brazil.; 9 Centro Universitário Christus, Fortaleza CE, Brazil.; 10 Universidade Federal do Ceará, Fortaleza CE, Brazil.

**Keywords:** Social Class, Neurodevelopmental Disorders, Child Poverty, Classe Social, Transtornos do Neurodesenvolvimento, Pobreza Infantil

## Abstract

The development of the human nervous system makes up a series of fundamental and interdependent events involving birth, growth, and neuronal maturation, in addition to the positive or negative selection of synapses of these neurons that will participate in the composition of neural circuits essential to the activity of the nervous system. In this context, where environment and social relationships seem to be relevant markers for neurodevelopment, advanced neuroimaging techniques and behavioral assessment tools have demonstrated alterations in brain regions and cognitive functions among children developing in low or high socioeconomic status environments. Considering the aspects mentioned, this review aimed to identify the importance of socioeconomic status in children’s brain development, seeking to identify what are the impacts of these factors on the morphological and physiological formation of the nervous system, allowing a greater understanding of the importance of environmental factors in neurodevelopmental processes.

## RESUMO

O desenvolvimento do sistema nervoso humano compõe uma série de eventos fundamentais e interdependentes envolvendo o nascimento, crescimento e maturação neuronal, além da seleção positiva ou negativa de sinapses desses neurônios que participarão da composição de circuitos neurais essenciais à atividade do sistema nervoso. Nesse contexto, em que o ambiente e as relações sociais parecem ser marcadores relevantes para o neurodesenvolvimento, técnicas avançadas de neuroimagem e ferramentas de avaliação comportamental têm demonstrado alterações em regiões cerebrais e funções cognitivas em crianças que se desenvolvem em ambientes de baixo ou alto nível socioeconômico. Considerando os aspectos mencionados, esta revisão teve como objetivo identificar a importância do *status* socioeconômico no desenvolvimento cerebral infantil, buscando identificar quais são os impactos desses fatores na formação morfológica e fisiológica do sistema nervoso, permitindo maior compreensão da importância dos fatores ambientais nos processos de neurodesenvolvimento.

## INTRODUCTION

It is a fact that neurodevelopment does not occur independently, as environmental factors and social connections that an individual experiences throughout his or her life significantly impact the development of cognitive and social skills^1^. In the same way, poverty is a plural marker and is linked to structural and functional differences in various areas of the brain^2^.

The development of the human nervous system makes up a series of fundamental and interdependent events involving birth, growth, and neuronal maturation, in addition to the positive or negative selection of synapses of these neurons that will participate in the composition of neural circuits essential to the activity of the nervous system^3^. As a constituent part of this system, the brain assumes the most complex functions, and the higher brain regions responsible for cognition experience a prolonged development that covers all growth periods from embryogenesis to adolescence^4,5^. This longitudinal characteristic of neurodevelopment^4^, associated with environmental factors that interfere with neuroplasticity mechanisms^5^, mediates the susceptibility of these events, contiguous to the varying conditions of socioeconomic status (SSE) during childhood^4^.

SSE is based on a multidimensional analysis^1^, and is broadly composed of family income, parental education level, and area deprivation^6^. The influence that is generated by the environment on SSE includes social and cultural factors, bordering associations and possible interferences in scientific papers that relate this component to some object of study^7^.

However, it is suggested that these brain changes associated with SSE modulate specific neurocognitive systems, acting differently in each one of them, through the activation of regions relevant to the performance of certain tasks^8^. Morphologically, punctuated alterations are observed as to volume and prolonged thickening in brain areas, changes in cortical and subcortical regions, apparent slower development of brain activity and changes in the distribution of white and gray matter, in addition to revealing reduced hemispheric specialization for language processing and less efficiency in functional network organization^6,9,10^ in children with lower SSE.

Considering the aspects mentioned, this review aims to identify the importance of SSE in children’s brain development, seeking to identify the impacts of these factors on the morphological and physiological formation of the nervous system, allowing a greater understanding of the importance of environmental factors in neurodevelopmental processes.

## METHODS

The present article reports a literature review that examined the impact of child poverty on brain development by analyzing scientific articles published between 2012 and 2022 in the United States National Library of Medicine (PubMed) and Science Direct databases. The review process involved applying three sets of criteria. Firstly, titles that did not mention the impact of child poverty on brain development, articles outside of the research period from 2012 to 2022, and articles not in English were excluded. Secondly, abstracts that were not relevant to the review’s focus were eliminated. Lastly, after reading the remaining articles, those that did not specifically relate to the theme of the review were excluded.

**Figure 1:**
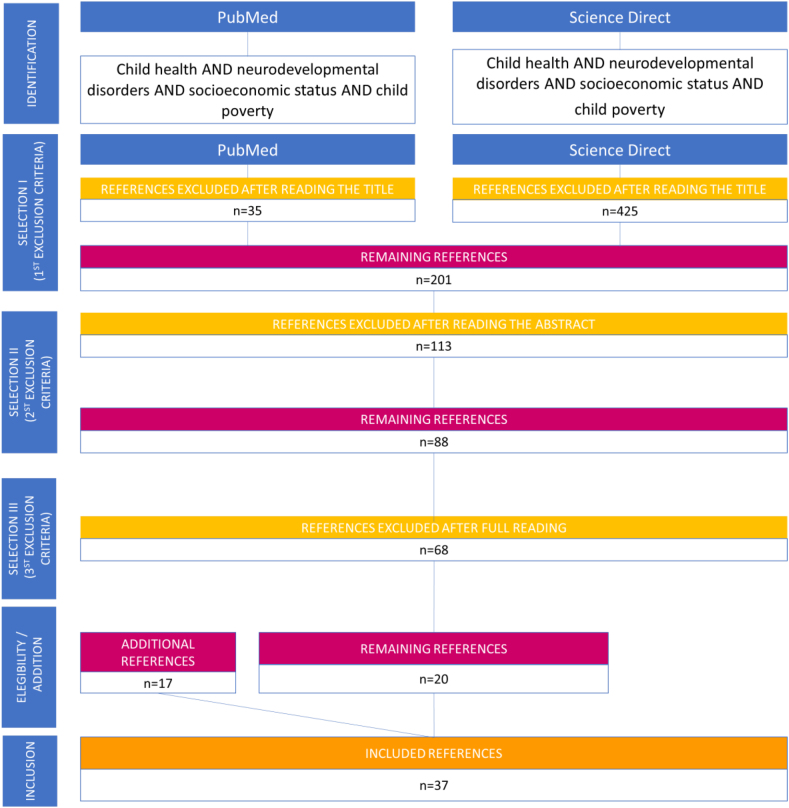
Flowchart of the article selection.

In PubMed, a search for “Child Health AND Neurodevelopmental Disorders AND Socioeconomic Status AND Child Poverty” yielded 60 articles, and 25 were selected after applying the first set of criteria. Similarly, a search for the same keywords in Science Direct resulted in 601 articles, from which 176 were selected. After applying the second set of criteria, 113 abstracts were excluded. The final set of criteria led to the exclusion of findings that were not related to the theme of the review, resulting in 37 selected articles originally published in English (see Figure 1 for a visual summary of the article selection process).

## RESULTS

Dumcke et al. revealed a notable increase in emotional and behavioral symptoms among children aged six to 13 years old who lived in closer proximity to garbage recycling sites. These sites were found to contain chemical, organic, and hospital waste, which were occasionally dispersed in the streets and came into contact with local residents^11^.

Dellefratte et al. and Dórea indicate that, during prenatal and childhood stages, exposure to pollutants can influence motor and cognitive development in children, leading to a predisposition for externalizing symptoms and behaviors indicative of attention-deficit/hyperactivity disorder (ADHD)^12,13^. The data reveals that exposure to benzene, toluene, ethylbenzene, and xylene (BTEX) air pollutants or other pollutants commonly linked to low SSE environments can elevate the probability of exhibiting behaviors suggestive of ADHD^12^.

The findings of a study by Rosen et al. exploring the connections between exposure to violence, cognitive stimulation, and the quality of the physical environment with SSE and the neural pathways of associative memory, signalized attention, and guided attention in children aged 60–75 months indicated that exposure to violence has a negative impact on associative memory during early childhood. Additionally, higher SSE was linked to improved performance in children’s memory-guided attention neural circuitry, which was attributed to the enhanced quality of their physical environment^14^.

Brady et al. revealed a correlation between residing in a neighborhood with a high rate of property crime during pregnancy and decreased neonatal functional connectivity within the anterior thalamic mode network^15^.

The results of a study by Singh and Ghandour show that low household SSE is associated with behavioral problems in children, with children from low-education and low-income families being 1.9–3.7 times more likely to suffer severe behavioral problems than those from more advantaged families, regardless of neighborhood conditions, family structure, and race/ethnicity^16^. In a separate study conducted in Ceará, Brazil, Correia et al. found that monthly family income, social class, and the level of food security of two- to six-year-old children belonging to low SSE families were strongly associated with developmental delays^17^.

Hair et al. conducted a multi-site longitudinal cohort study in the US, revealing that low SSE was associated with atypical development of brain structures potentially involved in academic development, such as the frontal lobe, temporal lobe, and hippocampus^18^. The study found that children exposed to lower financial resources had reduced gray matter volume and experienced greater maturational delays compared to less impoverished children.

In a meta-analysis by Taylor and Barch, a connection between reduced inhibitory control (IC) and lower academic performance was identified. The studies analyzed revealed significant detrimental effects of poverty, low IC, externalizing behaviors, internalizing behaviors, and school performance, with variations dependent on the social environment and duration of exposure to such conditions^19^.

Galler et al. highlighted the widely recognized detrimental effects of malnutrition on child neurodevelopment, demonstrating strong correlations between protein and micronutrient deficiencies and cognitive performance, intellectual abilities, intelligence quotient (IQ), behavior and attention deficits, and brain atrophy^20^.

A study by Ramphal et al. found that living in poverty can impact various stages of neurodevelopment, including nutrition, physical activity opportunities, and maternal psychological state, resulting in changes in neural network connectivity in fetuses^21^. The study reported a positive association between high cortisol levels, associated with stress and low SES, and changes in brain connectivity at birth. The findings suggest that poverty-related stressors may have lasting effects on fetal brain development and externalizing symptoms in early childhood^12^.

The study by Khoury et al. found that exposure to maternal withdrawal in infancy and borderline features in adulthood was associated with a reduction in hippocampal volume in adulthood, indicating the negative impact of early life stress on brain development and function^22^. Noble and Giebler revealed that children exposed to socioeconomic disadvantage exhibit slower growth of the hippocampal region. This structural discrepancy is suggested to increase as they age and may lead to further cognitive impairments^23^. The hippocampus, closely related to learning and memory capacity, is the most affected area when exposed to the neurotoxic effects of cortisol and stress^13^. Children living with socioeconomic disadvantage have slower growth of the hippocampal region, and this structural discrepancy may increase as they age^24^. Other brain regions, such as the amygdala, thalamus, and corpus striatum, which are related to emotion and reward processing, also appear to have links with SSE issues^13,24^.

According to Britto et al., poverty and high levels of stress during the puerperal and postnatal periods can weaken maternal bonds and contribute to weaknesses in care, leading to decreased breastfeeding and potential negative impacts on neurodevelopment^25^.

As reported by Noble and Giebler, cortical volume variations related to poverty and low SSE have been observed, with changes in cortical gray matter development, thickness, and volume in frontal and temporal cortices^23^.

According to Schneider et al., children exposed to constant stress, abuse, family instability, abuse, neglect, and witnessing domestic violence are at increased risk of developing schizophrenia^26^, borderline personality disorder, and a higher rate of suicide/self-mutilation as adults^13^.

## DISCUSSION

### Brain development and poverty environment

The differences in SSE among groups shape the multiple psychological processes. An example of the influence of SSE in this processing is the way people define themselves or how they perceive the world, and the reflex of this in the subjective psychological well-being^2^. The concept of psychological well-being may be interfered with by internal symptoms, such as the amplified allostatic load, or external symptoms, such as the persistence of helplessness and its nuances^17^. Similarly, an adequate definition of poverty status can be expressed by exposure to sub-optimal physical and psychosocial conditions, such as living in substandard housing and experiencing constant family turmoil^17^.

In this context, where environment and social relationships seem to be relevant markers for neurodevelopment, advanced neuroimaging techniques and behavioral assessment tools have demonstrated alterations in brain regions and cognitive functions among children developing in low or high SSE environments^14^. A robust body of recent research points to associations between brain development in childhood and this important marker of social position, i.e., SSE^19^. Socioeconomic variables, particularly family income, early childhood education, neighborhood quality, educational level, occupational and behavioral status of parents, maternal health status, pre- and postnatal infant feeding, and stress^8,14,23^, are possible risk factors for malnutrition, exposure to toxins, unstimulating environments, poor sleep quality, and poorer infant mental health^8,23^. In view of this, these vulnerabilities may impair child neurodevelopment by causing significant brain changes and generating cognitive deficits, affecting, for example, working memory and executive function^6,8^, in addition to favoring future psychopathological changes, language deficits, and sustained attention^9^.

Understanding the sequencing of an organism’s development, or ontogeny, as well as the actual genetic and epigenetic influences on neurodevelopment, has grown exponentially in recent decades. In essence, ontogeny transcribes the history and developmental stages of an organism throughout its life, from embryogenesis to the last day of life^18^. However, there are huge gaps in the knowledge about the structural and functional development of the human brain during early and late infancy.

Considering these precepts, it is known that the basis for the first stage of neurodevelopment starts a few weeks after conception and lasts for the first years of postnatal life. However, experiences throughout childhood may still exert a strong influence on the configuration, quantity and architecture of synapses and myelin sheath integrity, as both processes continue to develop after this first stage^18^.

After this phase, the second stage of development follows, where there is an expressive increase in cortical white matter, especially in the frontal, parietal and temporal lobes, which may last until the end of puberty (∼14 years of age)^18,27^. It is also during this period of puberty that the gray matter begins to regress, especially in the somatosensory areas. This process is more advanced in girls compared to boys by about two years^27^. The maturation of the human brain can continue until the age of 30. The early development of the prefrontal cortex during early childhood has important consequences and significantly affects how the first environments experienced by the child shape the development of key frontal circuits that are extremely relevant for complex cognitive skills^27^.

In light of these concepts, several studies have sought to present in a structured manner the possible causes of neurodevelopmental disorders in childhood, such as environmental factors, which are sometimes affected by socioeconomic inequalities. Low family income, for example, may be a predictor for the child to grow up in an environment with conditions that have a negative influence on his/her development. With these studies, we aimed to bring scientific basis that can contribute to practices that promote the physiological maturation of children.

Focusing on the geographical perspective, developing countries or low- and middle-income countries have higher poverty rates and, concomitantly, lower rates of child well-being associated with a higher prevalence of cognitive, socioemotional, and physical deficits^18,20^. This connection can also be seen locally, considering that the neighborhood in which the child lives due to a low SSE may be less safe and less structured in relation to environments for socialization, learning, and sanitary conditions. This generates a stressful environment^28^ and a greater risk of exposure to pathogens and neurotoxic substances that impact brain structure and function, leading, for example, to morphological changes such as the reduction of the hippocampal subfield, related to prenatal psychosocial stressors and exposure to polycyclic aromatic hydrocarbons^21^.

In a case-control study in Brazil, a developing country, the presence of emotional and behavioral difficulties was evaluated by means of the Strengths and Difficulties Questionnaire (SDQ), in children aged 6 to 13 years old who lived in low-income communities and resided less or more than 150 m away from recycling sites — which contained chemical, organic, and hospital waste, sometimes distributed in the street, in contact with the inhabitants. The data obtained showed accentuation of low SSE markers, with 46% of the children living closer to the centers being exclusively breastfed until the fourth month, 37% had no access to pre-school, 29.8% of the babies had smoking mothers, and 38% did not live with both parents at home. In this sense, the results showed greater emotional and behavioral symptoms in children living closer to the garbage recycling places^29^.

Still regarding this topic, other studies have shown that exposure to pollutants during stages such as prenatal and childhood may affect the motor and cognitive development of children and predispose to externalizing symptoms and behaviors suggestive of ADHD^30,31^. Exposure to BTEX air pollutants or other pollutants associated with low SSE increases the chances of these behaviors^12^. Recent research, therefore, sheds light on the influence of environmental changes on the neurodevelopment of an entire community sharing the same geographic location and, consequently, experiencing similar SSE effects.

Thus, sharing similar SSE markers is often one of the characteristics of more deprived communities. Several studies^15–17,32^ have shown significant socioeconomic influences of neighborhood and family on child health. The aspects present in these places that influence children’s health are socioeconomic deprivation, poor housing, crime, and lack of social amenities. Many of these problems can be significantly modified through social policies. The consequences of these conditions are evident in the results obtained by a survey in which children and adolescents living in more deprived neighborhoods or in neighborhoods characterized by poor housing, high rates of garbage and vandalism have significantly higher levels of behavioral problems and higher probability of suffering severe behavioral problems, which, even after controlling the families’ SSE does not culminate in the reduction of children’s externalizing symptoms^16^.

Furthermore, the poverty environment is often associated with higher exposure to violence. In a study of children aged 60–75 months, the relationship between exposure to violence, cognitive stimulation, and quality of the physical environment with SSE and the neural pathways of associative memory, signalized attention, and guided attention was observed. The results showed that exposure to violence negatively influences associative memory in early childhood, believed to be due to a mechanism related to chronic stress and increased glucocorticoids resulting in neurotoxic effects on the hippocampus. Furthermore, higher SSE was associated with better child performance in the neural circuitry of memory-guided attention due to increased quality of the physical environment, which tends to provide a more stimulating environment for the child^14^. A pertinent limitation of this study is that few children have an SSE close to the poverty line, keeping a sample with a relatively high average SSE, potentially masking greater effects of poverty on these neural circuits.

Moreover, the prenatal environment can also trigger modifications in the epigenome of the differentiating cell, leading to changes in organ structure and function^32^. In this regard, there are findings indicating that living in a neighborhood with a high rate of property crime during pregnancy was related to weaker neonatal functional connectivity between the anterior pattern mode network of the thalamus^15^. However, further studies are needed to identify the long-term behavioral impairments arising from these changes.

As far as the family environment is concerned, the health and bonding status of parents can be shaken by their SSE. In situations of financial difficulties, parents are likely to develop psychiatric disorders, such as anxiety and depression, and to generate conflicts among themselves, fostering a hostile and stressful environment for the child, in which beneficial parenting practices, such as time and effort dedicated to caring for the offspring, are reduced, and harmful parenting practices, such as neglect, violence, and repression, are expressed^32^. These situations, therefore, stimulate the appearance of emotional and behavioral disorders in these children.

**Figure 2:**
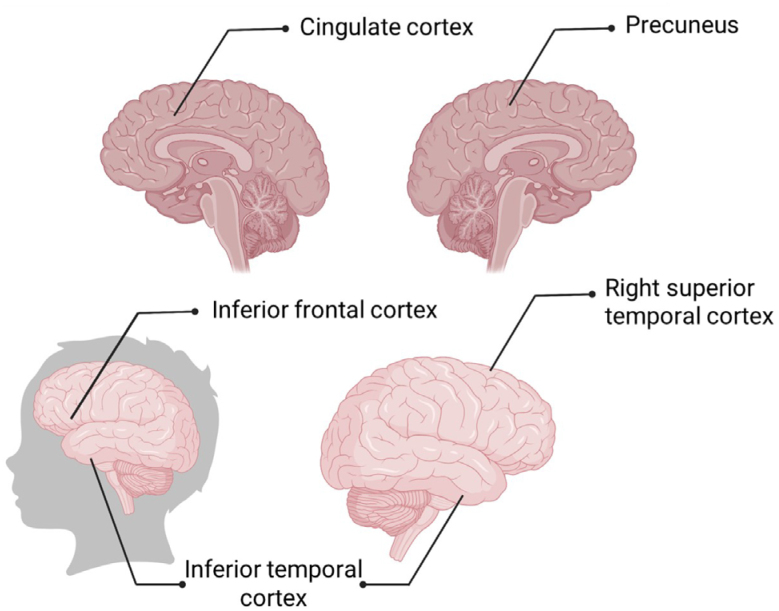
Areas of vulnerability on the poverty brain.

Further narrowing the relationship with the environment, low household SSE has also been associated with behavioral problems in children^16^. Regardless of neighborhood conditions, family structure, and race/ethnicity, children from low-education and low-income families were 1.9–3.7 times more likely to suffer severe behavioral problems than children from more advantaged families^16^. Monthly family income, social class, and the level of food security of two- to six-year-old children belonging to low SSE families in Ceará, Brazil, was strongly associated with developmental delays^17^. The difficulties of an impoverished upbringing reduce the surface area of some parts of the cortex more than others. The affected regions (Figure 2) participate in various forms of mental processing, such as language, perception, executive functions, and spatial abilities.

Finally, it is understood that neurodevelopment is indeed affected by the environment, which, when containing adversities such as violence, crime, high levels of pollutants, low family income, and other examples mentioned above, bring consequences to the development of children. The outcomes are diverse: eventual behavioral problems^16^, changes in the formation of the nervous system in utero^15^, developmental delays^17^, and behaviors suggestive of ADHD^12^. However, outside the family environment the low SSE also echoes, bringing about, for example, decreased academic performance^19^.

### Disparities in socioeconomic status and academic impact

The impact of SSE on academic achievement has become relevant in recent years. It is questioned whether discrepancies in academic life are the product of inferences to an individual in an environment of vulnerabilities (low SSE, malnutrition, and unsafe home settings) or whether the results are the product of social selection, where the genetic underpinnings of academic performance lead to disparities in SSE^23^. Recent studies from large cohorts^19,20,32^ have shown that early environmental experience has a strong impact on physical, cognitive, social, and emotional appearance domains^32^ in childhood, which together may converge on functional academic performance. Given the inquiry, we recognize in the scientific literature a strong association between SSE in childhood and academic performance.

Hair et al. aimed to delineate irregular patterns of brain development in the face of family poverty and impairments in academic performance^18^. The multi-site longitudinal cohort study in the US followed 389 typical children and adolescents aged four to 22 years over a six-year period with sociodemographic and neuroimaging data. The sample reflected the demographic disposition of race/ethnicity and income. The results showed that there is evidence that low SSE may be a relevant factor in the manifestation of childhood human capital by relating it to atypical development of brain structures that possibly mediate academic development, in addition to greater vulnerability to environments early in life. The structures observed in the study were the frontal lobe (attention, cognitive flexibility, emotional regulation), temporal lobe (memory, language, assignment of meaning to words), and hippocampus (information processing, long-term memories).

In this construct, the lower the financial resources children are exposed to, the lower the volume of gray matter in addition to the maturational delay in relation to less poor children. From a developmental science perspective, there is a legitimate interest in investigating the strict relationship between neural bases and educational phenomena, and what environmental elements carry this relationship. Researchers have identified that there is a clear association between early life poverty and the performance of inhibitory control (IC)^19,27^. A sensitive period for this function occurs during early childhood, reporting that its maturation process may be unique and sensitive to stimuli/environments^19^ during this time. In this context, it was evidenced that moving to poorer neighborhoods significantly influenced externalizing symptoms within the classroom^19^. Taylor and collaborators found that the more exposure to resource scarcity, the slower the growth of IC^19^. A meta-analysis investigating IC in the context of poverty, using a method indexed by parent/teacher reports and behavioral measures, found results that linked lowered IC to lower academic performance^19^.

The IC is defined as a component of the executive functions, which authorizes the suppression or not of automatic responses, and is fundamental for thoughts and behaviors aimed at effective goals, planning, anticipation, decision-making, and social interactions, which are higher order functions located in the cortex, considered to be the most relevant neural structure in terms of cognitive skills. Other investigations reveal associations between parental SSE and the brain structure of children and adolescents^27^, including cortical thickness and gray matter volume^1^, especially in frontal and temporal regions^23^ that support areas of language, attention, memory, executive functioning, and emotions^23^, a process closely linked to learning ability, information retention, and academic performance. Therefore, there is supported evidence in the literature that IC is a key factor in proximal and distal academic impact.

Plural studies report pronounced deleterious effects between poverty, low IC, externalizing behaviors (hyperactivity, aggressiveness, impulsivity)^5,19^, internalizing (anxiety, stress, depression) and school performance. In these research studies, the effects have variations according to the social environment, conditions and time of exposure of the child to them. A relevant element in this context is insecure attachment, according to Bowlby’s studies on Attachment Theory, insecurity in the attachment unfolds in deficits in self-regulation, for example, which leads to educational failure. Caring for a child involves multiple elements, such as good health, proper nutrition, safety, and security^27^, that are elements of a propelling gear into adulthood. Secure bonds with caregivers are predictors for good emotional connectivity, the ability to build secure relationships, and positive self-esteem later in life. Yet, vulnerable environments that compromise the performance of parents and caregivers propose a shortened favorable condition, contributing to physical, cognitive, emotional, and behavioral impairments.

In the dimension of food insecurity^20^, the deleterious effects of malnutrition on child neurodevelopment are already widely accepted; there is strong evidence that protein and micronutrient deficiencies affect cognitive performance, intellectual abilities, IQ, behavior and attention deficits, and brain atrophy, items that are mostly relevant in academic performance. However, gaps remain in this research topic, such as the lack of resources and technology to advance studies in target countries, where malnutrition rates are prevalent in the population.

Furthermore, it is apparent that malnutrition manifests itself within an environment with greater adversities and disadvantages, and, it seems, may compromise research findings, as there are still no reports of an assertive measurement between early malnutrition and brain structure and function. Even so, the findings allow researchers to infer that the lack of macronutrients and micronutrients resulting from a poor diet promotes damage to neurodevelopment and, consequently, losses in school life from early childhood to elementary school and throughout life.

### Poverty, stress and brain development

In general, SSE is measured in the main approaches considering a correlation between the factors that can directly or indirectly influence the individual’s growth and development. The elements to be evaluated to establish SSE include the parents’ level of education, occupational prestige, family income^21,23,28^ and the possibility of access to health care^21^. By considering SSE as a determinant of human development processes, attempts have been made to establish correlations between this measure of social position and the incidence of its effects on brain development^21^, an essentially noble proposal, considering that it is an important functional component for biopsychosocial well-being and, ultimately, survival.

Thus, living in poverty is a stressor^25^ that may affect the different stages of neurodevelopment^21^. Poverty extends its effects on nutrition, physical activity possibilities, and maternal psychological state, raising cortisol levels, a hormone that has been associated with changes in neural network connectivity in fetuses^21^. Studies have shown that the hippocampus was the most affected area when exposed to the neurotoxic effects of cortisol and stress, especially in the prenatal phase and early post-uterine life, with evidence of morphological changes in the volume of this region^22^, leading to cognitive and neurological deficits^33^. Furthermore, low SSE and stress factors are agents of intrauterine growth restriction and also seem to favor premature birth, factors that would increase the risk for inadequate neurodevelopment; however, the mechanisms of this interference are still unclear^25^.

It is important to highlight that the hippocampus is a nervous system structure that is closely related to learning and memory capacity^23^. The development of the hippocampus occurs rapidly, especially in the first two years of life, reaching its maximum volume around nine to 11 years of age^22^. In this sense, children living with socioeconomic disadvantage have a slower growth of the hippocampal region, and this structural discrepancy may increase as the years go by^23^. However, the hippocampus is not the only structure that is related in the main studies. The development of regions such as the amygdala, thalamus, and corpus striatum, which are related to emotion and reward processing, also seem to have links with SSE issues^22,23^.

Nevertheless, as previously discussed, neurodevelopment is a longitudinal event that accompanies the individual for a long period of time^4,5^. This makes this process fragile from the point of view of environmental influences, and thus, SSE also has a preponderant role throughout the postnatal period and infancy. The vulnerabilities surrounding newborns and infants seem to assume an essential character in the scope of this discussion. When considering the puerperal and postnatal period in women, poverty and high levels of stress tend to weaken maternal bonds.

It is evident that the elements that contribute to low SSE, such as the need to return to work early, the deprivation of financial resources, and violence, among others, can significantly increase the weaknesses in care^25^ and, consequently, in the child’s development and growth. Thus, maternal breastfeeding becomes the target of these determinants and can be affected in such a way as to generate losses in neurodevelopment. Infant nutrition by exclusive breastfeeding has been shown to influence the IQ tests of evaluated minors^25^. On the other hand, when stopped early, it is a potential risk agent prevalent, for example, in children with intellectual disability^34^.

Furthermore, the effects of exposure to poverty and, consequently, to stress factors in early childhood endure and sometimes accompany the advancing age of the individual^35^. A decrease in orbitofrontal volume, for example, was observed up to 25 years later in individuals who experienced poverty early in life. And even when there were improvements in socioeconomic factors during the course of childhood, the disturbances were maintained^23^. Cortical volume variations have also been widely addressed and expressed in some studies with respect to poverty and low SSE. However, cortical volume has proven to be an imprecise assessment tool, since it is an indirect result consisting of the surface area and the thickness of the cortex, but the studies using it are usually consistent and show that there is diversity in the development of the cortical gray matter, such as the thickness and volume of the frontal and temporal cortices^23^, regions that house essential functions, as previously described.

On the other hand, the prefrontal cortex and its neural networks allow communication with other brain areas and are highlighted in studies about the influence of stress on neurodevelopmental processes, since it is a critical area for early learning^35^. The alterations in cerebral cortex connectivity, caused by the consequences of low SSE and poverty, also negatively influence behavioral inhibition and result in difficulty to control impulses, besides favoring hyperactivity, aggressiveness, and other externalizing symptoms^21^.

Neuropsychiatric disorders, especially those already mentioned in this study, have been the subject of studies in the field of neuroscience, which seek to show whether a low SSE can influence the predisposition for them to occur. It is known that the child who lives in an environment of constant stress, with mistreatment, family instability, abuse, neglect, and witnessing domestic violence, has an increased risk of developing schizophrenia^26^, borderline personality disorder, and a higher rate of suicide/self-mutilation as an adult^22^.

In conclusion, despite efforts to cover the most diverse associations between SSE and risk factors for neurodevelopment, the studies used reflect diverse methodologies that focus on different age groups, locations, and assessment scales, which raises the levels of limitations in their results.

It should, however, be noted that there is evidence of a correlation between the environment-related issues already elucidated in this review and their influences that sometimes orchestrate changes in neurodevelopment. Therefore, in the search to establish specific markers, their interference in brain development, and incidences in different age groups, as well as to obtain greater results of interventions aimed at preventing the impacts of low SSE, it becomes imperative to conduct new studies that can further elucidate the important question that was proposed, if money matters and how much it matters.
